# Effects of respiratory rate on venous-to-arterial CO_2_ tension difference in septic shock patients undergoing volume mechanical ventilation

**DOI:** 10.1186/s40001-020-00402-9

**Published:** 2020-03-17

**Authors:** Zhixiang Guo, Yapeng Wang, Chao Xie, Guofang Hua, Shenglin Ge, Yuedong Li

**Affiliations:** 1 Department of Cardiovascular Surgery, The First Affiliated Hospital of Anhui Medical University, No. 218 Jixi Road, Hefei, 230022 Anhui China; 2 Department of Cardiac Intensive Care Unit (CICU), The First Affiliated Hospital of Anhui Medical University, No. 218 Jixi Road, Hefei, 230022 Anhui China; 3 Department of Critical Care Medicine, The 901st Hospital of the Joint Logistics Support Force of PLA, No. 424 Changjiang West Road, Hefei, 230000 Anhui China

**Keywords:** Sepsis, Septic shock, Venous-to-arterial carbon dioxide tension difference, Volume-controlled ventilation, Respiratory rate

## Abstract

**Background:**

To explore the effects of the respiratory rate (RR) on the venous-to-arterial CO_2_ tension difference (gapCO_2_) in septic shock patients undergoing volume mechanical ventilation.

**Methods:**

Adult patients with septic shock underwent volume mechanical ventilation between October 2015 and October 2016. RR was started at 10 breaths/min, and 2 breaths/min were added every 60 min until 16 breaths/min was reached. At every point, central venous and arterial blood gas measurements were obtained simultaneously.

**Results:**

In this study, gapCO_2_ induced by hyperventilation significantly increased, while the central venous carbon dioxide pressure (PvCO_2_) and the partial pressure of CO_2_ (PaCO_2_) in arteries decreased. The decreasing trend of the PaCO_2_ was more obvious than that of the PvCO_2_. HCO_3_^−^ and ctCO_2_ were markedly decreased, when the RR was increased (*P* < 0.05). Central venous oxygen saturation (S_cv_O_2_) had a decreasing trend between 14 (77.1 ± 8.3%) and 16 (75.2 ± 8.7%) breaths/min; however, the difference was not significant.

**Conclusions:**

In septic patients undergoing ventilation, respiratory alkalosis induced by hyperventilation caused an increase in the gapCO_2_. Clinicians should cautiously interpret the gapCO_2_ in hemodynamically stable ventilated septic shock patients and its relationship with low cardiac output and inadequate perfusion.

## Background

The current consensus and guidelines for the hemodynamic management of severe sepsis and septic shock advocate clinicians to regard the use of lactate clearance and central venous oxygen saturation (S_cv_O_2_) normalization as tissue perfusion endpoints [1–3]. Abnormally high S_cv_O_2_ values have been related to higher mortality in septic shock patients [4], while lactate clearance has proved to be as beneficial as the S_cv_O_2_ in the hemodynamic management of sepsis. However, when clinicians face the uncertainty of a high lactate value, they have to identify whether it indicates persistent hypoperfusion or whether it will eventually normalize. Therefore, normalized S_cv_O_2_ values and lactate values do not rule out persistent hypoxia and hypoperfusion during initial resuscitation.

A few authors have reported that the central venous-to-arterial CO_2_ difference (gapCO_2_) demonstrated prognostic value in different conditions [5–8] and have recommended that the gapCO_2_ combined with the S_cv_O_2_ and lactate values should be used to rule out patients with persistent global hypoperfusion [3]. Under steady states of both CO_2_ production and oxygen consumption, the gapCO_2_ was observed to be increased in parallel with the reduction in cardiac output [9]. However, spontaneous breathing and hyperventilation may reduce the partial pressure of CO_2_ in arteries (PaCO_2_) and prevent the CO_2_ stagnation-induced rise in central venous carbon dioxide pressure (PvCO_2_). Mallat et al. demonstrated that acute hyperventilation provoked a significant increase in gapCO_2_ and oxygen consumption (VO_2_) [10]. To date, little is known about the gapCO_2_ and respiratory rate (RR) in septic shock patients undergoing volume mechanical ventilation. Therefore, in this study, we investigated the underlying effect of the RR on the gapCO_2_ in septic shock patients undergoing volume mechanical ventilation.

## Materials and methods

### Patients

This observational and prospective study was performed at a single, mixed medical and surgical adult intensive care unit (ICU) with 14 beds at 105 Hospital of Chinese People’s Liberation Army from October 2015 to October 2016. The study was approved by the ethics committee of the 105 Hospital of Chinese People’s Liberation Army. Informed consent was obtained from each subject.

The study included 17 patients within 24 h of septic shock onset. The diagnosis of septic shock was determined according to the Second International Consensus of Definitions for Sepsis and Septic Shock [11]. As part of the routine management of septic shock in our ICU, all patients underwent volume mechanical ventilation with a fractional inspired oxygen level (FiO_2_) no greater than 65%, using an 840 Ventilator System.

Sedation with propofol and analgesia with remifentanil were provided so that patients without spontaneous breathing could be included in the study. The inclusion criteria were oliguria, mottled skin [12], central venous oxygen saturation (S_cv_O_2_) > 65% and lactate level < 4 mmol/L after achieving adequate intravascular volume and adequate mean arterial pressure (MAP) > 65 mmHg, as recommended by the Surviving Sepsis Campaign [1]. The exclusion criteria were pregnancy, COPD, age less than 18 years, unstable hemodynamic condition (change of vasoactive drug dosage or fluid administration within 60 min before the protocol), high blood lactate levels (> 4 mmol/L) after adequate resuscitation, and uncontrolled tachyarrhythmia (heart rate > 140 beats/min).

### Measurements

Demographic data, septic shock etiology, and acute physiology and chronic Health evaluation (APACHE) II [13] and sequential organ failure assessment (SOFA) scores were obtained on the day of enrollment. Blood gas analysis was performed using a blood gas analyzer (ABL 900 Radiometer, Copenhagen, Denmark). S_cv_O_2_ was determined in a sample taken from the central venous catheter with the tip (confirmed by X-ray) in the superior vena cava near or at the right atrium. The gapCO_2_ was calculated as the difference between the central PvCO_2_ tension and PaCO_2_ tension.

### Study protocols

Ventilation parameters and vasopressor drugs were kept constant during the 60-min period prior to the measurements being taken. After the baseline measurements were taken, an RR of 10 breaths/min was started, and 2 breaths/min were added every 60 min until 16 breaths/min was attained. For each procedure, all parameters were measured. The study ended when arterial hypotension (MAP < 60 mmHg), tachycardia (heart rate > 150 beats/min), acute atrial fibrillation, or changes in the ST segment of the electrocardiogram occurred.

### Statistical analysis

Statistical analysis was performed using SPSS 10.0 software. A p value (2-tailed) of 0.05 or less was considered significant. Continuous variables were presented as mean ± standard deviation (SD) if normally distributed, or as median if otherwise, and compared using Wilcoxon–Mann–Whitney test. Comparisons of hemodynamic indexes and blood gas in accordance with the RR from 10 to 16 breaths/min were performed by the Bonferroni method.

## Results

**Fig. 1:**
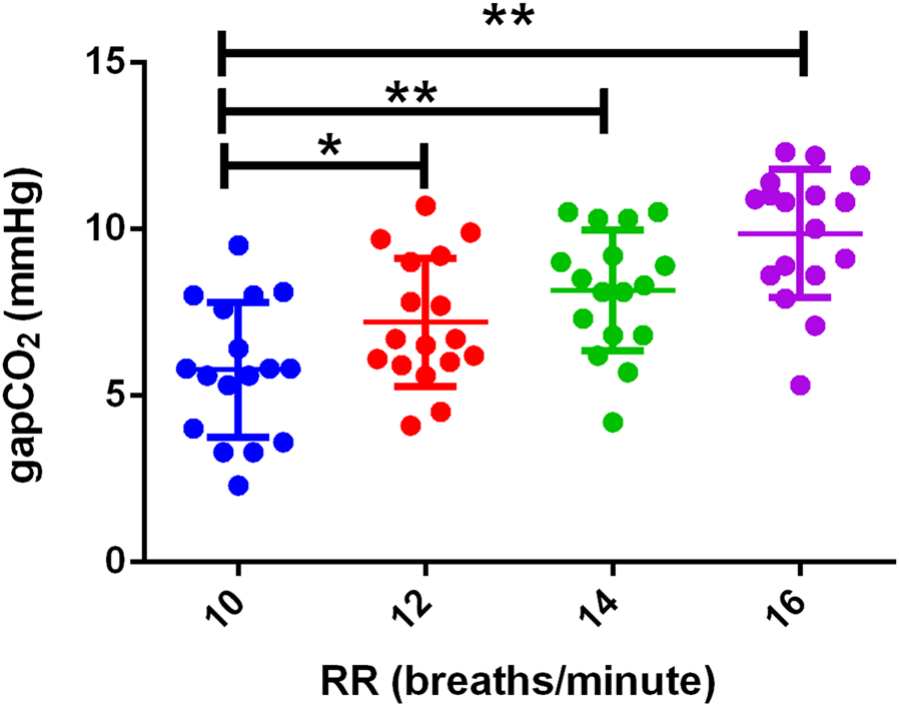
GapCO_2_ at different respiratory rates. ^*^P < 0.05 and ^**^P < 0.01 versus 10 breaths/mi

**Fig. 2:**
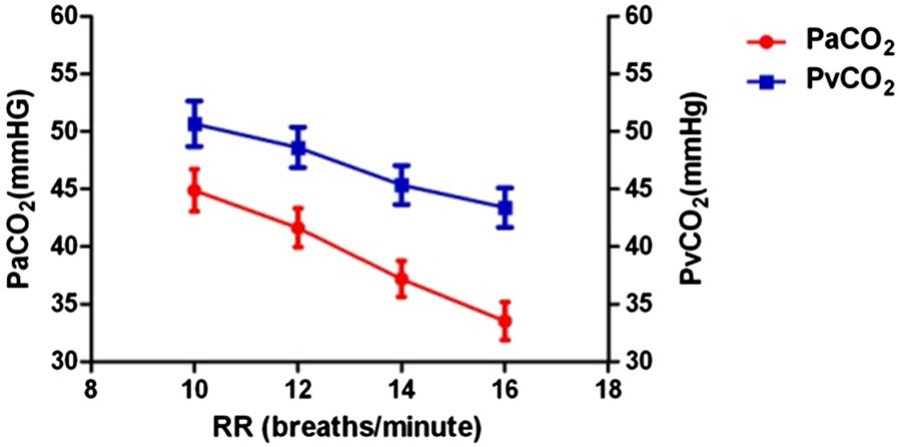
PaCO_2_ and PvCO_2_ at different respiratory rates

**Fig. 3:**
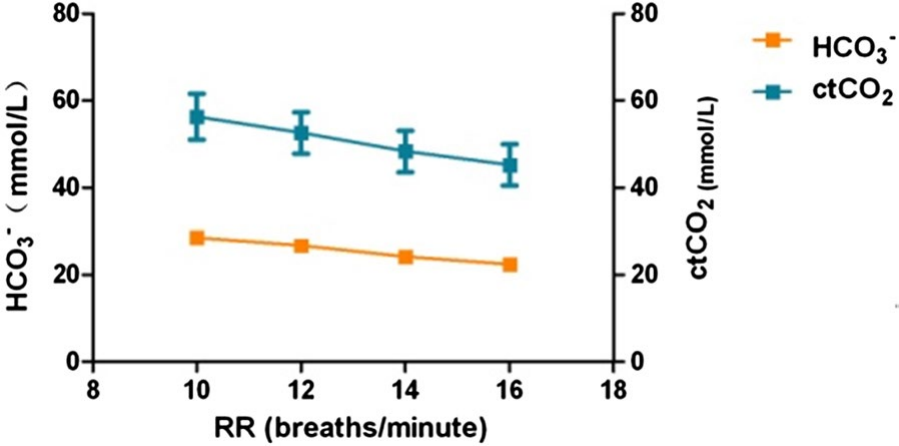
HCO_3_^−^ and ctCO_2_ at different respiratory rates

**Fig. 4:**
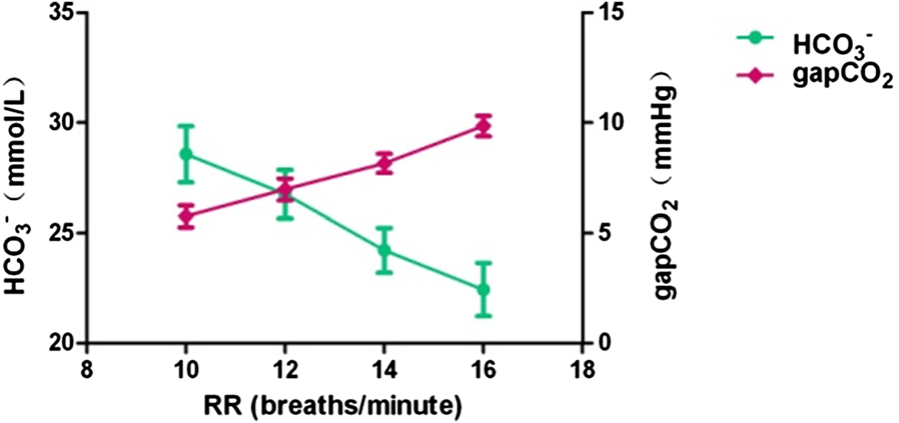
HCO_3_^−^ and gapCO_2_ at different respiratory rates

Basic characteristics of the 17 patients are presented in Table 1. All hemodynamic effects following the change in RR are listed in Table 2. Blood pressure, heart rate, lactate level, and BE changes were not statistically significant from 10 to 16 breaths/min. Hyperventilation induced a significant RR-dependent increase in the gapCO_2_, which was related to the combined trend of decreases in PaCO_2_ and PvCO_2_ (Figs. 1 and 2). Changes in HCO_3_^−^ were paralleled by a significant decrease in ctCO_2_ (Fig. 3). S_cv_O_2_ significantly increased with increasing RR from 10 to 14 breaths/min, but had a decreasing trend from 14 to 16 breaths/min. Hypocapnia provoked by hyperventilation significantly decreased from 10 to 16 breaths/min, while the gapCO_2_ exhibited an opposite trend (Fig. 4).

**Table 1:** Patients characteristics

Demographics	All patients (*n* = 17)
Age (years)	69 ± 11
APACHE II	20 ± 5
SOFA score at admission	7 ± 2
ICU mortality, *n* (%)	6
Lactate levels (mmol/L)	4.03 ± 0.2
CVP (cmH_2_O)	12.5 ± 3.6
Mottled skin, *n* (%)	6
Source of infection, *n* (%)	17
Pneumonia	8
Abdominal infection	5
Urinary tract infection	3
Mediastinal infection	1

Data are presented as mean ± SD or absolute values (%)

APACHE II, acute physiology and chronic health evaluation; SOFA, sepsis-related organ failure assessment; ICU, intensive care unit; CVP, central venous pressure

**Table 2:** Hemodynamic, blood gas, and metabolic parameters during different RRs after achieving adequate intravascular volume

Parameters	Level 1 (RR = 10)	Level 2 (RR = 12)	Level 3 (RR = 14)	Level 4 (RR = 16)
HR, beats/minute	95 ± 19	98 ± 17	97 ± 17	97 ± 15
MAP, mmHg	84 ± 9.5	84 ± 10.9	83 ± 9.8	86.9 ± 11.7
pH	7.35 ± 0.078	7.38 ± 0.092^*^	7.42 ± 0.059^**^	7.43 ± 0.078^**^
PaCO_2_, mmHg	44.9 ± 7.60	41.6 ± 6.92^**^	37.2 ± 6.42^**^	33.5 ± 6.80^**^
PaO_2_, mmHg	114.1 ± 24.96	125.0 ± 24.67^*^	137.6 ± 26.86^**^	144.3 ± 27.57^**^
SaO_2_, %	98.9 ± 1.15	99.1 ± 0.91	99.2 ± 0.74	99.1 ± 0.90
Lactate, mmol/L	1.78 ± 0.95	1.89 ± 1.29	1.98 ± 0.72	2.15 ± 2.06
BE	0.59 ± 9.48	0.42 ± 6.51	0.70 ± 4.59	0.84 ± 4.40
K^+^, mmol/L	4.3 ± 0.9	4.3 ± 0.7	4.2 ± 0.7	4.4 ± 0.6
HCO_3_^−^, mmol/L	28.58 ± 5.22	26.76 ± 4.56^*^	24.23 ± 4.20^*^	22.44 ± 4.99^*^
ctCO_2_, mmol/L	56.32 ± 21.9	52.62 ± 19.7^*^	48.33 ± 19.5^**^	45.21 ± 19.6^**^
PcvCO_2_, mmHg	50.66 ± 8.09	48.61 ± 7.21^**^	45.35 ± 6.99^**^	41.43 ± 12.50^**^
S_cv_O_2_, %	73.1 ± 7.7	75.3 ± 7.0	77.1 ± 8.3^**^	75.2 ± 8.7^*^
gapCO_2_, mmHg	5.76 ± 2.02	7.19 ± 1.93^*^	8.16 ± 1.81^**^	9.85 ± 1.92^**^

Data are presented as mean ± SD or absolute values (%)

MAP, mean arterial pressure; PaCO_2_, partial pressure of carbon dioxide in artery; PcvCO_2_, central venous carbon dioxide pressure; gapCO_2_, central venous-to-arterial carbon dioxide difference; S_cv_O_2_, central venous oxygen saturation

^*^P < 0.05 compared with RR of 10 breaths/min, ^**^P < 0.01 compared with RR of 10 breaths/min. Multiple comparisons were used to adjust for the Bonferroni method

## Discussion

Septic patients with respiratory failure suffer myocardial systolic and diastolic dysfunction [14]. Mechanical ventilation plays a critical role in these patients. At present, the gapCO_2_ has been considered an evaluation of tissue hypoperfusion and has been recommended as a guide for resuscitation in septic patients [5, 6]. However, it is unknown whether the clinical significance of the gapCO_2_ also applies to septic patients with mechanical ventilation. Therefore, we explored the effects of the respiratory rate on the gapCO_2_ in septic shock patients undergoing volume mechanical ventilation and found a significant difference in the gapCO_2_ induced by hyperventilation.

When the respiratory rates increased with the tidal volume unchanged, the total minute ventilation and alveolar ventilation, as well as the PaO_2_, improved. The PaCO_2_ decreased because excessive ventilation enabled the increased expiration of CO_2_. Our results showed that the PcvCO_2_ and PaCO_2_ decreased with an increasing respiratory rate, and this difference was statistically significant. However, the decreasing trend of the PaCO_2_ was more obvious than that of the PcvCO_2_. Consequently, the difference in the gapCO_2_ values when the RR varied from 10 to 16 breaths/min was significant. We consider that the following physiological factors can explain this phenomenon.

The combination of oxygen and hemoglobin can promote the release of CO_2_. Furthermore, CO_2_ more easily combines with hemoglobin at lower oxygen saturation, which is a phenomenon called the Haldane effect [15]. Clearly an increase in alveolar ventilation increases CO_2_ excretion and alveolar PaO_2_. Increased alveolar ventilation causes respiratory alkalosis with immediate changes in hydrogen ion concentrations and bicarbonate concentrations. There are transcellular shifts with H ions coming out cells into the plasma and shifts of potassium from the plasma into cells. Renal compensation with bicarbonate excretion takes hours. Nevertheless, respiratory alkalosis can cause peripheral vasoconstriction and decreased flow through the various vascular beds. This is probably not a uniform effect in all vessels and may or may not occur in patients with sepsis. However, if blood flow decreases but metabolism is unchanged, then the CO_2_ content of venous blood should increase, leading to a higher gapCO_2_. If patients with sepsis have disordered cellular metabolism, then the production CO_2_ may decrease, which in turn results in a lower gapCO_2_. Furthermore, the depression of oxidative adenosine triphosphate (ATP) production also led to pulmonary vasoconstriction [16]. Nitric oxide synthesis defect has shown to be another cause [17]. As a consequence, the reducing trend of the CO_2_ partial pressure of venous blood is less marked than that of arterial blood. This has been demonstrated by other scholars as well [18]. The increase in alveolar ventilation increases CO_2_ excretion and should increase the alveolar PaO_2_ provided other factors stay the same. Increased alveolar ventilation causes respiratory alkalosis with immediate changes in hydrogen ion concentrations and bicarbonate concentrations. There should be transcellular shifts with hydrogen ion coming out cells into the plasma and shifts of potassium from the plasma into cells. Respiratory alkalosis can cause peripheral vasoconstriction and decreased flow through various vascular beds. This is probably not a uniform effect in all vessels and may or may not occur in patients with sepsis. However, if blood flow decreases but metabolism is unchanged, then the CO_2_ content of venous blood should increase, resulting in a gapCO_2_. If patients with sepsis have dysfunctional cellular metabolism, then the production CO_2_ may decrease, which would result in a lower gapCO_2_. This likely varies in the vascular bed from tissue to tissue. CO_2_ binding hemoglobin may not have an important effect in these experiments.

When vasoconstriction is caused by hypocapnia, poor oxygen delivery may add tissue oxygen extraction [19]. Hence, when patients are in hypocapnia provoked by hyperventilation, the CO_2_ partial pressure of the venous blood does not obviously decrease compared with the CO_2_ partial pressure of the arterial blood. For instance, Khambatta et al. reported that mechanically ventilated dogs undergoing 2 h of hyperventilation had 20% CO_2_ production and 25% O_2_ consumption [20]. In this study, the S_cv_O_2_ significantly increased with the RR increase from 10 to 14 breaths/min, but there was no significant decreasing trend in S_cv_O_2_ between 14 and 16 breaths/min, which was consistent with a previous report [21]. Moreover, it is widely accepted that the gapCO_2_ depends on measurements from the superior vena cava (SVC) but does not reflect measurements from the inferior vena cava (IVC) and tissues draining into the IVC [22].

Patients with septic shock lack an effective circulating blood volume, which leads to systemic anoxia and metabolic acidosis. Acidosis and excess CO_2_ can cause mitochondrial dysfunction [23, 24]. Metabolic acidosis and respiratory alkalosis due to excessive hyperventilation further reduce the function of mitochondria oxidative phosphorylation, which may impact CO_2_ production. In summary, our data suggested that in septic patients with mechanical ventilation, respiratory alkalosis induced by hyperventilation enabled the increase of gapCO_2_. Clinicians should be cautious when interpreting the gapCO_2_ as low cardiac output and inadequate perfusion in hemodynamically stable ventilated septic shock patients.

## Conclusions

However, there were some limitations in our paper. In this study, we used a central venous sample to measure the gapCO_2_ instead of using a mixed venous sample, despite the latter having a better correlation. We cannot state the relationship between the gapCO_2_ and cardiac output, as we did not include the indexes of cardiac function in this study. We included fewer patients than other studies, and the included patients suffered from different etiological factors, so the results from this study should be further confirmed in the future.

## Data Availability

The datasets used and/or analyzed during the current study are available from the corresponding author on reasonable request.
